# Comment on “Polygonati Rhizoma Prevents Glucocorticoid‐Induced Growth Inhibition of Muscle via Promoting Muscle Angiogenesis Through Deoxycholic Acid” by Shi et al.

**DOI:** 10.1002/jcsm.70074

**Published:** 2025-09-24

**Authors:** Zhihao Lei

**Affiliations:** ^1^ Department of Statistics and Data Science Cornell University Ithaca New York USA

To the Editor,

We read with interest the article by Shi et al. [1], which reports that *Polygonati Rhizoma* (PR) extract and a fructose/glucose mixture (MFG) attenuate dexamethasone‐induced muscle growth inhibition by promoting “muscle angiogenesis through deoxycholic acid (DCA)” [1]. While the idea of a gut–muscle axis linking a traditional herbal treatment to muscle perfusion is intriguing, we have significant concerns about the evidence and interpretation. Specifically, the proposed mechanistic chain (PR → gut microbiota → DCA → TGR5 → cAMP/PKA/CREB → VEGF → angiogenesis → muscle growth) is largely speculative, the experiments are underpowered, statistical support is weak, the “active component mimic” MFG is undefined, and correlations are overstated as causation. We outline our main points below.

First, the mechanistic cascade proposed by Shi et al. is inadequately supported. The authors claim MFG “upregulated VEGFs expression and promoted muscle angiogenesis via the TGR5/cAMP/PKA/pCREB pathway” [1], implying a causal link from gut‐derived DCA to muscle VEGF. However, the data are purely correlative. No functional experiments (e.g., DCA supplementation or TGR5 inhibition) were performed to prove any step. Emerging research on the “gut–muscle axis” warns that microbiota‐muscle associations are not proof of mechanism. For example, a recent review notes that although gut microbes correlate with sarcopenia risk, “whether the microbiome is the cause of the disease or simply a mediator … is debatable” [2]. More broadly, microbiome studies are now emphasizing the need to move beyond associations to demonstrate causality [3]. In Shi et al., PR/MFG treatments were found to raise intestinal DCA and abundance of *Collinsella aerofaciens* (*p* = 0.096) [1], and muscle VEGF gene signatures were higher. But without intervening on DCA or TGR5, these observations remain hypothesis‐generating at best. As noted by the authors themselves, muscle angiogenesis involves complex, multilevel regulation [1]; assuming that a change in one bile acid automatically drives the entire VEGF → angiogenesis program is an overreach.

Second, the sample sizes are very small. Key in vivo comparisons involve only *n* = 8 (AEPR vs. control), *n* = 10 (MFG vs. control), or *n* = 7 (DEX vs. DEX + MFG) mice [1]. Such low *n* values are likely underpowered. The ARRIVE guidelines explicitly warn that “sample sizes that are too small … produce inconclusive results” [4]. Low power not only risks missing real effects but, paradoxically, also inflates the estimated effect size and false positive rate [4, 5]. Button et al. showed that typical small‐ N neuroscience studies have very low power, meaning that even “a statistically significant result is less likely to reflect a true effect” [5]. Shi et al. do not report any power calculations or justification of group sizes. With n ≈ 7–10, even a *p* < 0.05 finding should be viewed with caution. It is also unclear whether the multiple t‐tests used were corrected for multiple comparisons, further risking spurious significance. In sum, the statistical power of these experiments appears insufficient to robustly test the complex hypotheses advanced.

Third, several reported p‐values are marginal and need careful interpretation. For example, Shi et al. note that MFG “increased the abundance of *C. aerofaciens* as measured by DNA concentration” (0.80 vs. 0.24 pg./μL) but report *p* = 0.096 [1]. This is above the conventional 0.05 threshold, so it should not be deemed statistically significant. Yet the text implies a biological difference. Similarly, the DCA increase (log_2 intensity 25.41 vs. 22.69) is reported with *p* < 0.05 [1], but with *n* = 7 and no mention of variability of DCA levels, the robustness is questionable. In small‐sample studies, p‐values near 0.05 are notoriously fickle [5]. One review emphasizes that underpowered experiments often yield significant p‐values that overestimate the true effect [4]. In the absence of confidence intervals or replication, the borderline *p*‐values in [1] do not inspire confidence. We suggest that the authors should either increase sample sizes or employ stricter statistical criteria (e.g., lower α or adjusting for multiple tests) before drawing strong conclusions.

Fourth, the definition of MFG as an “active component mimic” of PR is vague and unsupported [1]. PR extract contains a mixture of sugars, polysaccharides, glycosides, etc., as is common for herbal preparations. The authors state that PR is “rich in fructose” [1] and therefore use a 50:50 fructose/glucose mix (MFG) to represent its effect. But they provide no evidence that fructose and glucose are the relevant bioactive factors. In fact, PR has many reported constituents (e.g., saponins, polysaccharides) with unknown activity. Standard pharmacological practice would require isolating and chemically characterizing the active compound(s) in PR and then testing them individually [6]. As one review notes, lack of quality control in herbal products leads to “inconsistent levels of active compounds … and variable therapeutic effects” [6]. Without any chemical analysis or reference standards, treating MFG as if it were PR's “active ingredient” is speculative. It remains entirely possible that some other PR component, not represented by simple sugars, mediates the effects. We therefore find the reliance on MFG without validation to be a major weakness in attributing PR's action to dietary sugars.

Finally, the manuscript tends to interpret correlations as causal evidence. For instance, the authors conclude that MFG “mitigates muscle growth inhibition by modulating gut microbiota and enhancing muscle angiogenesis” [1]. Yet the data only show that MFG *associates* with higher intestinal DCA and muscle mass. As noted above, the causal link (DCA → TGR5 → VEGF→growth) is inferred but not tested. In microbiome studies, it is well recognized that associations alone cannot establish mechanism [3]. To demonstrate that DCA drives muscle effects would require additional experiments, such as treating mice with DCA directly or blocking its receptor (TGR5) and observing the outcome on VEGF and muscle. Without such data, phrases like “DCA‐activated TGR5 pathway” are premature. We therefore urge caution: the observations in [1] are hypothesis‐generating but do not prove the proposed causal chain.

In conclusion, while Shi et al. present an intriguing hypothesis, the current data do not firmly substantiate it. We recommend that future studies employ larger cohorts with appropriate power calculations, include biochemical validation of proposed active compounds, and use interventional designs to test causality along the pathway. For example, confirming that exogenous DCA or blocking TGR5 alters muscle angiogenesis would greatly strengthen the claims. Such steps would help ensure that the purported PR → gut→muscle mechanism is supported by clear causal evidence. We believe that pursuing these rigorous approaches will advance this line of inquiry more convincingly.

## Conflicts of Interest

The author declares no conflicts of interest.

## References

[1] S. Shi , R. Li , Y. Han , et al., “ *Polygonati Rhizoma* Prevents Glucocorticoid‐induced Growth Inhibition of Muscle via Promoting Muscle Angiogenesis Through Deoxycholic Acid,” Journal of Cachexia, Sarcopenia and Muscle 16, no. 3 (2025): e13853, 10.1002/jcsm.13853. 40522031 PMC12168230

[2] D. J. Barry , S. S. X. Wu , and M. B. Cooke , “The Relationship Between Gut Microbiota, Muscle Mass and Physical Function in Older Individuals: A Systematic Review,” Nutrients 17, no. 1 (2024): 81, 10.3390/nu17010081. 39796514 PMC11722951

[3] M. Basic , D. Dardevet , P. M. Abuja , et al., “Approaches to discern if microbiome associations reflect causation in metabolic and immune disorders,” Gut Microbes 14, no. 1 (2022): 2107386, 10.1080/19490976.2022.2107386. 35939623 PMC9361767

[4] N. Percie du Sert , A. Ahluwalia , S. Alam , et al., “Reporting animal research: explanation and elaboration for the ARRIVE guidelines 2.0,” PLoS Biology 18, no. 7 (2020): e3000411, 10.1371/journal.pbio.3000411. 32663221 PMC7360025

[5] K. S. Button , J. P. A. Ioannidis , C. Mokrysz , et al., “Power failure: why small sample size undermines the reliability of neuroscience,” Nature Reviews. Neuroscience 14, no. 5 (2013): 365–376, 10.1038/nrn3475. 23571845

[6] H. Wang , Y. Chen , L. Wang , Q. Liu , S. Yang , and C. Wang , “Advancing Herbal Medicine: Enhancing Product Quality and Safety Through Robust Quality Control Practices,” Frontiers in Pharmacology 14 (2023): 1265178, 10.3389/fphar.2023.1265178. 37818188 PMC10561302

